# Overcoming the matched-sample bottleneck: an orthogonal approach to integrate omic data

**DOI:** 10.1038/srep29251

**Published:** 2016-07-12

**Authors:** Tin Nguyen, Diana Diaz, Rebecca Tagett, Sorin Draghici

**Affiliations:** 1 Wayne State University, Department of Computer Science, Detroit, 48202, Michigan, USA; 2 Wayne State University, Department of Obstetrics and Gynecology, Detroit, 48202, Michigan, USA

## Abstract

MicroRNAs (miRNAs) are small non-coding RNA molecules whose primary function is to regulate the expression of gene products via hybridization to mRNA transcripts, resulting in suppression of translation or mRNA degradation. Although miRNAs have been implicated in complex diseases, including cancer, their impact on distinct biological pathways and phenotypes is largely unknown. Current integration approaches require sample-matched miRNA/mRNA datasets, resulting in limited applicability in practice. Since these approaches cannot integrate heterogeneous information available across independent experiments, they neither account for bias inherent in individual studies, nor do they benefit from increased sample size. Here we present a novel framework able to integrate miRNA and mRNA data (vertical data integration) available in independent studies (horizontal meta-analysis) allowing for a comprehensive analysis of the given phenotypes. To demonstrate the utility of our method, we conducted a meta-analysis of pancreatic and colorectal cancer, using 1,471 samples from 15 mRNA and 14 miRNA expression datasets. Our two-dimensional data integration approach greatly increases the power of statistical analysis and correctly identifies pathways known to be implicated in the phenotypes. The proposed framework is sufficiently general to integrate other types of data obtained from high-throughput assays.

High-throughput technologies for gene expression profiling, such as DNA microarray or RNA-Seq, have transformed biomedical research by allowing for comprehensive monitoring of biological processes. A typical comparative analysis of expression data, e.g. patients versus healthy samples, generally yields a set of genes that are differentially expressed (DE) between the conditions. These sets of DE genes contains the genes that are likely to be involved in the biological processes responsible for the disease. However, such sets of genes are usually insufficient to reveal the underlying biological mechanisms. In addition, due to inherent bias and batch effects present in individual studies, independent experiments studying the same disease often yield completely different lists of DE genes, making interpretation extremely difficult^1^,^2^,^3^.

In order to translate these lists of DE genes into a better understanding of biological phenomena, researchers have developed a variety of knowledge bases that map genes to functional modules. Depending on the amount of information that one wishes to include, these modules can be described as simple gene sets based on a function, process or component (e.g., the Molecular Signatures Database MSigDB^4^), organized in a hierarchical structure that contains information about the relationship between the various modules, as found in the Gene Ontology^5^, or organized into pathways that describe in details all known interactions between the various genes that are involved in a certain phenomenon. Pathway databases include: the Kyoto Encyclopedia of Genes and Genomes (KEGG)^6^,^7^, Reactome^8^, and Biocarta (www.biocarta.com).

Analysis techniques have been developed to help interpret such sets of DE genes. The earliest approaches use Over-Representation Analysis (ORA)^9^,^10^ to identify gene sets that have more DE genes than expected by chance. The drawbacks of this type of approach include that: (i) it only considers the number of DE genes and completely ignores expression changes; (ii) it assumes that genes are independent, which they are not; and (iii) it ignores the interactions between various modules. Functional Class Scoring (FCS) approaches, such as Gene Set Enrichment Analysis (GSEA)^11^ and Gene Set Analysis (GSA)^12^, have been developed to address some of the issues raised by ORA approaches. The main improvement of FCS is the observation that small but coordinated changes in expression of functionally related genes can have significant impact on pathways. Both FCS and ORA approaches can be used with gene sets, ontologies, or pathways. However, these approaches do not account for the hierarchical structure of pathways or interactions between genes. Topology-based approaches, which fully exploit all the knowledge about how gene interact as described by pathways, have been developed more recently. The first such techniques were ScorePAGE^13^ for metabolic pathways and the Impact Analysis^14^ for signaling pathways.

Non-coding RNAs, especially microRNAs (miRNAs) have come into the spotlight more recently. Data describing observed and predicted interactions between miRNA and mRNA is accumulating rapidly in several databases, such as miRTarBase^15^, miRWalk^16^, starBase^17^, and TargetScan^18^. In addition, miRNA expression platforms, datasets and analysis tools^19^,^20^ have become more and more prevalent.

Two of the most widely used approaches to include miRNA expression data for the purpose of pathway analysis are Micrographite^21^ and PARADIGM^22^. Micrographite^21^ is a topology-aware pathway analysis approach that is able to integrate sample-matched miRNA and mRNA expression. PARADIGM^22^ uses a probabilistic graphical model (PGM) to integrate information of different data types, which may include mRNA and miRNA.

The first drawback of these tools for integrating miRNA and mRNA is that they need sample-matched data. In other words, these tools require both data types to be available for each individual patient. This reduces their practical availability since sample-matched data is relatively rare and difficult or expensive to obtain. Therefore, the vast amount of available expression data, both mRNA and miRNA, is not fully utilized.

The second drawback is that these methods are unable to exploit heterogeneous information available across independent studies. Therefore, they are not able to address the inevitable bias inherent in individual studies. It would be tremendously beneficial if all datasets associated with a given condition could be analyzed together because of the increased power expected to be associated with the much larger number of measurements in the combined dataset. Large public repositories such as Gene Expression Omnibus^23^,^24^, The Cancer Genome Atlas (cancergenome.nih.gov), ArrayExpress^25^, and Therapeutically Applicable Research to Generate Effective Treatments (ocg.cancer.gov/programs/target) store thousands of datasets, within which there are independent experimental series with similar patient cohorts and experiment design. Expression data, mRNA as well as miRNA, are particularly prevalent in public databases, such that some disease conditions are represented by half a dozen studies or more.

The process of combining sample-matched data of different types is referred to as *vertical* integrative analysis, while that of combining multiple unmatched studies using the same data type is referred as *horizontal* meta-analysis^26^. Thus, they are considered *orthogonal* classes of data integration. For microarray data, the method proposed by Rhodes *et. al*.^27^ was one of the earliest *horizontal* approaches to combine multiple microarray datasets, using Fisher’s method. Since then, other sophisticated approaches have been proposed for the integration of multiple gene expression datasets, on both the gene and pathway levels^28^,^29^,^30^. The majority of these meta-analysis approaches work by combining the p-values obtained from individual gene expression datasets. However, they typically do not try to account for the data heterogeneity, attributed to batch effects, patient heterogeneity, and disease complexity, responsible for expression changes across different sources.

Here we propose a framework that is able to integrate unmatched miRNA and mRNA data obtained from many independent laboratories. While validated in the context of pathway analysis, the framework can be modified to adapt to other domains or applications. This framework is not meant to compete with any existing approach, but to serve as a bridge between *horizontal* and *vertical* data integration. Each building block or technique of our pipeline can be easily substituted for by any other similar technique to suit the purpose of future analysis.

We illustrate the new framework using 15 mRNA and 14 miRNA datasets related to two human diseases, colorectal cancer and pancreatic cancer. They were generated by independent labs, for different sets of patients. For both conditions, the new framework is able to identify pathways relevant to the phenotypes. We demonstrate that the accuracy is obtained only by integrating the data in both directions (horizontal and vertical).

To the best of our knowledge, this is the first article that describes an orthogonal meta-analysis. Our results suggest that orthogonal classes of integrative techniques can be further combined to unravel the underlying mechanisms of complex diseases. With vast databases of various data types being made available, this framework is expected to be widely applicable because of its relaxed restrictions on the data being integrated.

## Methods

The classical pathway analysis begins by considering a comparison between two conditions, e.g. disease versus healthy. Evidence for differential gene expression can be provided by any technique such as fold change, t-statistic, Kolmogorov-Smirnov statistic, or perturbation factor. These statistics are then compared against the null distribution to determine how unlikely it is for the observed differences between the two conditions to occur by chance, thereby producing a ranked list of DE genes. After this hypothesis testing is done at the gene level, the next step is hypothesis testing at the pathway level producing a ranked list of impacted pathways. In summary, the input of a classical pathway analysis method includes: (i) a pathway database, and (ii) a gene expression dataset. The output is a list of pathways ranked according to their p-values.

Similarly, the input of the new approach includes: (i) a pathway database, (ii) a database of miRNA-mRNA interactions, (iii) multiple gene expression datasets, and (iv) multiple miRNA expression datasets. Each dataset is obtained from an independent study of the same disease. Here we describe a framework that transforms the new problem into the classical pathway analysis problem.

**Figure 1:**
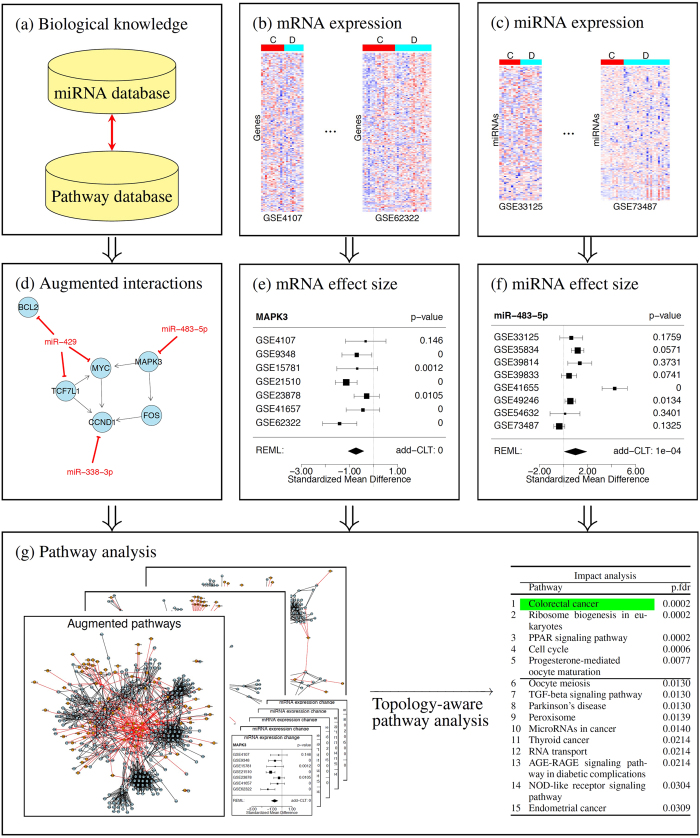
Overall pipeline of the proposed framework. The input consists of (i) a pathway database and a miRNA database including known targets (panel a), (ii) multiple mRNA expression datasets (panel b), and (iii) multiple miRNA expression datasets (panel c). Each expression dataset consists of two groups of samples, e.g. disease versus control. The framework first augments the signaling pathways with miRNA molecules and their interactions with coding mRNA genes (panel d). It then calculates the standardized mean difference and its standard error in each expression dataset. The summary size effect across multiple datasets for each data type are then estimated using the REstricted Maximum Likelihood (REML) algorithm (panels e,f). Similarly, the p-value for differential expression is calculated for each dataset and then combined using the additive method (add-CLT). The augmented pathways, the combined p-values, and the estimated size effects then serve as input for ImpactAnalysis, which is a topology-aware pathway analysis method (panel g).

Figure 1 illustrates the pipeline of our framework, for the case of colorectal cancer. Panel (a) represents the biological knowledge obtained from public databases: pathway information and miRNA targets. Panel (b) shows a set of gene expression datasets obtained from independent studies, coming from different laboratories. For this example, we have 7 datasets (GSE4107, GSE9348, GSE15781, GSE21510, GSE23878, GSE41657, and GSE62322), all related to the same disease, colorectal cancer. Each dataset consists of two groups of samples: disease (group D) and control/healthy (group C). Panel (c) represents a set of miRNA expression datasets (GSE33125, GSE35834, GSE39814, GSE39833, GSE41655, GSE49246, GSE54632, and GSE73487), also from colorectal cancer. Similar to gene expression datasets, each miRNA dataset consists of disease and control samples. The data provided in panels (a,b,c) serve as the input for our framework.

Pathways in public databases are typically described as graphs, where nodes are genes and edges are interactions between genes. In the first step, we extend the existing pathways with additional interactions between miRNAs and mRNAs. Panel (d) shows a part of the pathway *Colorectal cancer*, where blue nodes are genes and red nodes are miRNAs. The black arrow-headed lines represent activation while the red bar-headed lines represent inhibition. For example, *hsa-miR-483-5p* is known to suppress the expression of *MAPK3* and therefore an inhibition relationship is added between the two nodes in the pathway. All pathways are extended to include the known miRNA-mRNA interactions. The next step is to estimate the expression changes of each node (gene, miRNA) under the effects of the disease.

Panel (e) shows the expression changes and the p-values for one gene in the mRNA data, across several datasets. In this case, the *MAPK3* gene is used as an example. In the forest plot shown in this panel, each horizontal line represents the expression change in each study. The small black box in each line shows the standardized mean difference (SMD) and the segment shows the confidence interval of SMD. We use the standardized mean difference instead of the raw difference because the independent studies measure the expression in a variety of ways (different platforms, sample preparation, etc.). The number on the right side of each line is the p-value of the test for differential expression, using the modified t-test provided in the limma package^31^.

As shown in the figure, the SMD and p-value of a gene vary from study to study. We use the REstricted Maximum Likelihood (REML) algorithm^32^,^33^,^34^,^35^ to estimate the central tendency of SMD. We also use the add-CLT method^28^ to combine the independent p-values. Likewise, we compute the estimated SMDs and p-values for miRNA datasets (panel f).

The augmented pathways, the combined p-value, together with the estimated size effect then serve as input for classical pathway analysis. In this work, we use Impact Analysis, which is a topology-aware pathway analysis method, to calculate the p-value for each augmented pathway (panel g).

### Standardized mean difference for each gene

Consider a study composed of two independent groups, and suppose we wish to compare their means for a given gene. Let 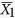 and 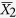 represent the sample means for that gene in the two groups, *n*_1_ and *n*_2_ the number of samples in each group, and *S*_*pooled*_ the pooled standard deviation of the two groups. The pooled standard deviation and the standardized mean difference (SMD) can be estimated as:


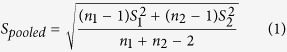



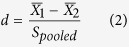


The estimation of the standardized mean difference described in Equation (2) is often called Cohen’s *d*^36^,^37^. The variance of Cohen’s *d* is given as follows:


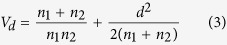


In the above equation, the first term reflects uncertainty in the estimate of the mean difference, and the second term reflects uncertainty in the estimate of *S*_*pooled*_. The standard error of *d* is the square root of *V*_*d*_. We note that Cohen’s *d*, which is based on sample averages, tends to overestimate the population effect size for small samples. Let *n* be the degrees of freedom used to estimate *S*_*pooled*_, i.e. *n* = *n*_1_ + *n*_2_ − 2. The corrected effect size, or Hedges’ *g*^38^, can be computed as follows:


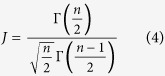



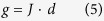


where Γ is the gamma function. In this work, we use Hedge’ *g* as the standardized mean difference (SMD) between disease and control groups for each gene/miRNA.

### Random-effects model and REML

Consider a collection of *m* studies where the effect size estimates, *y*_1_, …, *y*_*m*_ have been derived from a set of studies, each of them modeled as in Equation (5). A fixed-effects model would assume that there is one true effect size which underlies all of the studies in the analysis, such that all differences in observed effects are due to sampling error. However, this assumption is implausible since it cannot account for heterogeneity between studies^32^,^33^,^34^,^35^.

In contrast, the random-effects model allows for variability of the true effect. For example, the effect size might be higher (or lower) in studies where the participants are older, or have a healthier lifestyle compared to others. The random-effects model assumes that each effect size estimate can be decomposed into two variance components by a two-stage hierarchical process^33^,^39^,^40^. The first variance represents the variability of the effect size across studies, and the second variance represents the sampling error within each study. We can write the random-effects model as:


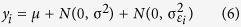


where *μ* is the central tendency of the effect size, *N*(0, *σ*^2^) represents the error term by which the effect size in the *i*^*th*^ study differs from the central tendency *μ*, and 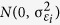 represents the sampling error.

The derivation and formulation of the REstricted Maximum Likelihood (REML) algorithm has been described in the literature^33^,^41^,^42^,^43^. The log-likelihood function for Equation (6) is given by:


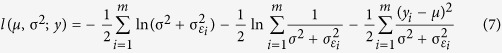


The REML estimators of 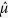 and 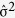 are then computed by iteratively maximizing the log-likelihood. In our framework, we calculate 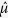 for each node (mRNA and miRNA) of the extended pathways. The estimated *overall effect size* 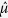 and the *combined p-value* of individual genes and miRNAs serve as the input for Impact Analysis.

### Combining independent p-values

We first briefly recap some classical methods for combining independent p-values. Next, we describe the additive method^28^,^44^,^45^,^46^ that is used to combine p-values for each mRNA and miRNA molecule in our framework.

Fisher’s method^47^ is the most widely used method for combining independent p-values. Considering a set of *m* independent significance tests, the resulting p-values *P*_1_, *P*_2_, …, *P*_*m*_ are independent and uniformly distributed on the interval [0, 1] under the null hypothesis. The random variables *X*_*i*_ = −2ln*P*_*i*_ (*i* ∈ {1, 2, …, *m*}) follow a chi-squared distribution with two degrees of freedom (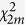). Consequently, the log product of *m* independent p-values follows a chi-squared distribution with 2*m* degrees of freedom. We note that if one of the individual p-values approaches zero, which is often the case for empirical p-values, then the combined p-value approaches zero as well, regardless of other individual p-values. For example, if *P*_1_ → 0, then *X* → ∞ and therefore, *Pr*(*X*) → 0 regardless of *P*_2_, *P*_3_, …, *P*_*m*_.

Stouffer’s method^48^ is another classical method that is closely related to Fisher’s. The test statistic of Stouffer’s method is the sum of p-values transformed into standard normal variables, divided by the square root of *m*. Denoting *ϕ* as the standard normal cumulative distribution function, and *p*_*i*_ (*i* ∈ [1..*m*]) the individual p-values that are independently and uniformly distributed under the null, the z-scores are calculated as *z*_*i*_ = *ϕ*^−1^ (1 − *p*_*i*_). By definition, these z-scores follow the standard normal distribution. The summary statistic of Stouffer’s method 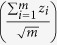 also follows the standard normal distribution under the null hypothesis. Similar to Fisher’s method, the combined p-values approach zero when one of the individual p-values approaches zero.

The additive method^28^,^44^,^45^,^46^,^49^ uses the sum of the p-values as the test statistic, instead of the log product. Consider the p-values resulting from *m* independent significance tests, *P*_1_, *P*_2_, …, *P*_*m*_. Let the sum of these p-values, 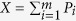 (*X* ∈ [0, *m*]), be the new random variable. *X* is known to follow the Irwin-Hall distribution^45^,^46^ with the following probability density function (pdf):


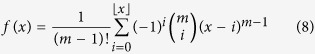


when *m* is large, some addends will be too small or too large to be stored in the memory. This leads to a totally inaccurate calculation when *m* passes a certain threshold, depending on the number of bits used to store numbers on the computer. For this reason, a modified version of the additive method, named add-CLT, was proposed^28^.

Let *Y* represent the average of p-values: 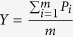 (*Y* ∈ [0, 1]). Since 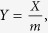 the probability density function (pdf) and the corresponding cumulative distribution function (cdf) of *Y* can be derived using a linear transformation of *X* as follows:


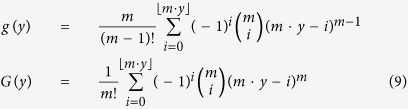


The variable *Y* is the mean of *m* independent and identically distributed (i.i.d.) random variables (the p-values from each individual experiment), that follow a uniform distribution with a mean of 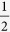 and a variance of 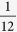. From the Central Limit Theorem^50^, the average of such *m* i.i.d. variables follows a normal distribution with mean 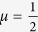 and variance 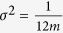, i.e. 
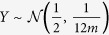
 for sufficiently large values of *m*. The transition from the additive method to the Central Limit Theorem takes place at the *m* ≥ 20 threshold.

In this work, we use the add-CLT method described above to combine the p-values calculated from the modified t-test (limma package).

### Graphical representation of augmented pathways

Here we give the formal description of the pathway augmentation process. Let *P* = (*V, E*) be the graphical representation of the pathway we want to extend with miRNA-mRNA interactions. *V* is the set of vertices (genes) while the directed edges in *E* represent the interactions between genes in the pathway. Each interaction consists of an ordered pair of vertices and the type of interaction between the pair, i.e. *E* = {(*x*_*i*_, *y*_*i*_), *r*_*i*_} where *x*_*i*_, *y*_*i*_ ∈ *G* (gene set) and *r*_*i*_ is the type of relation between *x*_*i*_ and *y*_*i*_, such as *activation, repression, phosphorylation, etc*. Topology-based pathway analysis methods, such as Impact Analysis, use interaction types to weigh the edges or to set the strength of signal propagation along the paths in a pathway.

From the miRNA database, we get a set of miRNAs and their targets. Let us denote *Z* as the set of known miRNAs, *ζ* ∈ *Z* is one miRNA, and *t*(*ζ*) is the set of known targets for the miRNA *ζ*. The augmented pathway of *P* = (*V, E*) is denoted as P* = (V*, E*) and is constructed as follows:


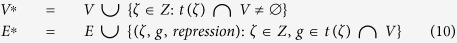


In other words, if a miRNA *ζ* targets a gene *g* that belongs to the pathway, we add *ζ* to the pathway and then connect *ζ* with its targets in the pathway. By default, the interaction type of new edges is *repression*, which represents the translation blockage of miRNAs to mRNA. The interaction type can be changed to suit the interaction between the miRNA molecule and its targets. We extend all pathways in the pathway database using the formulation described in Equation (10). The R package mirIntegrator^51^ for pathway augmentation is available on Bioconductor website (www.bioconductor.org).

### Impact analysis of augmented pathways

The Impact Analysis method^14^,^52^ combines two types of evidence: (i) the over-representation of DE genes in a given pathway^9^,^10^, and (ii) the perturbation of that pathway, caused by disease, as measured by propagating expression changes through the pathway topology. These two aspects are captured, respectively, by the independent probability values, *P*_*NDE*_ and *P*_*PERT*_. Here we review the Impact Analysis formulation.

The first p-value, *P*_*NDE*_, is obtained using the hypergeometric model^9^,^10^, which is the probability of obtaining at least the observed number of differentially expressed genes. The second p-value, *P*_*PERT*_, depends on the identity of the specific genes that are differentially expressed as well as on the interactions described by the pathway. It is calculated based on the perturbation factor in each pathway. The perturbation factor of a gene, *PF*(*g*), is calculated as follows:


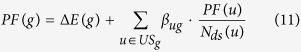


The first term represents the signed normalized expression change of the gene *g*, i.e. log standardized mean difference as shown in panels (e,f) of Fig. 1. The second term is the sum of perturbation factors of upstream genes, normalized by the number of downstream genes of each such upstream gene. The value of *β*_*ug*_ quantifies the strength of interaction between *u* and *g*. Here, *β*_*ug*_ = 1 for *activation* and *β*_*ug*_ = −1 for *repression*.

The above equation essentially describes the perturbation factor PF for a gene as a linear function of the perturbation factors of all genes in a given pathway. In the stable state of the system, all relationships must hold, so the set of all equations defining the impact factors for all genes form a system of simultaneous equations whose solution will provide the values for the gene perturbation factors *PF*_*g*_. The net perturbation accumulation at the level of each gene, *Acc*(*g*), is calculated by subtracting the observed expression change from the perturbation factor.


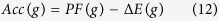


The total accumulated perturbation in the pathway is then computed as:


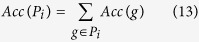


The null distribution of *Acc*(*P*_*i*_) is built by permutation of expression change. The p-value, *P*_*PERT*_, is then calculated by the probability of having values more extreme than the actually observed *Acc*(*P*_*i*_).

To compute *P*_*NDE*_ and *P*_*PERT*_, the following input is required: the graphical representation of the pathway, the combined p-value of each node of the graph, and the estimated overall standardized mean difference. In short, the graphical representation of the augmented pathways is provided in Equation (10), the p-value for each node of the augmented pathways is computed using Equation (9), and the expression change, Δ*E*(*g*), is estimated by iteratively maximizing the log-likelihood function in Equation (7). These two p-values, *P*_*NDE*_ and *P*_*PERT*_, are then combined to get a single p-value that represents how likely the pathway is impacted under the effect of the disease.

## Experimental Results

We analyzed a total of 1,471 samples from 29 public datasets for two human diseases, colorectal and pancreatic cancer. The datasets were generated in independent laboratories, from different individual tissue samples, and were run on different high-throughput platforms. The diseases were selected based on two criteria: (i) there are many publicly available miRNA and mRNA datasets, and (ii) there is a pathway specific to the disease (target pathway). The colorectal data consists of 7 mRNA and 8 miRNA datasets while the pancreatic data consists of 8 mRNA and 6 miRNA datasets. The processed data sets were downloaded directly from the Gene Expression Omnibus using the GEOquery package^53^. The data were rescaled using a log transformation if they were not already in log scale (base 2). The details of each dataset, such as the number of samples, tissues, and platforms, are reported in Table 1.

The databases used in our data analysis are KEGG for pathways, and miRTarBase for miRNAs. We downloaded 182 signaling pathways from KEGG version 76 (Dec-04-2015) by means of the R package ROntoTools^54^. We augmented these pathways with known miRNAs and their target interactions, downloaded from miRTarBase. For each mRNA/miRNA, we use the *modified t-test*, available in the limma package^31^, to test for differential expression of mRNA/miRNAs. We use *add-CLT*^28^ as the method to combine independent p-values. We then adjust the combined p-values for multiple comparisons using False Discovery Rate (FDR)^55^. For expression change, we use *Hedges’ g*^38^ as effect size, and the *REML* method^56^ to estimate the central tendency of effect sizes. Following convention, we only take into consideration mRNA/miRNAs having FDR-corrected combined p-values less than 5%. Among these significant genes, we choose mRNA/miRNAs that have the highest estimated SMD as differentially expressed, up to 10% of total measured mRNA/miRNAs. All the R scripts used for data processing, pathway augmentation, and analysis are available on demand from the authors.

For both diseases, we compare the orthogonal approach (ImpactAnalysis_I) with 5 other approaches: pathway-level meta-analysis (ImpactAnalysis_P), gene-level meta-analysis (ImpactAnalysis_G), plus the 3 meta-analysis approaches available in MetaPath package^29^,^30^. Since the input data sets consist of multiple studies, none of which are sample-matched, we are unable to perform pathway analysis using approaches that integrate matched mRNA and miRNA expression.

For pathway-level meta-analysis (ImpactAnalysis_P), we perform Impact Analysis on each mRNA expression dataset and then combine the independent p-values for each pathway. For example, if we have 7 mRNA datasets, we have 7 nominal p-values per pathway–one for each study. These 7 p-values are independent and thus can be combined using the add-CLT method to get one combined p-value. The final result is a list of 182 p-values for 182 signaling pathways. We then adjust the combined p-values for multiple comparisons using FDR.

For gene-level meta-analysis (ImpactAnalysis_G), we perform the modified t-test^31^ for each mRNA dataset and then combine the p-values. With 7 mRNA datasets, for example, each gene will have 7 independent p-values, which will be combined into one p-value. We also calculate the SMD and standard error of each gene in each study, then use the REML algorithm to calculate the overall effect size across the 7 studies. Finally, pathway analysis is performed on 182 KEGG pathways using the combined p-values and the estimated effect sizes, resulting in a list of pathways ranked according to their p-values. We then adjust the p-values of pathways for multiple comparisons using FDR.

The integrative approach (ImpactAnalysis_I) is similar to ImpactAnalysis_G, with the exception that ImpactAnalysis_I uses both mRNA and miRNA data. The meta-analysis is done on the mRNA/miRNA level and then the combined p-values and estimated effect sizes of mRNA/miRNAs serve as the input to the ImpactAnalysis.

MetaPath^29^,^30^ is a dedicated approach that performs meta-analysis at both gene (MetaPath_G) and pathway levels (MetaPath_P) with a GSEA-like approach, and then combines the results (MetaPath_I) to give the final p-value and ranking of pathways. MetaPath first calculates the t-statistic for each gene in each study. In MetaPath_G, these statistics are combined for each gene using maxP^57^. The combined statistics are then used to calculate enrichment scores for each pathway using a Kolmogorov-Smirnov test. In MetaPath_P, the pathway enrichment analysis is done first before meta-analysis. In MetaPath_I, the p-values of MetaPath_G and MetaPath_P are combined using minP^58^.

**Figure 2:**
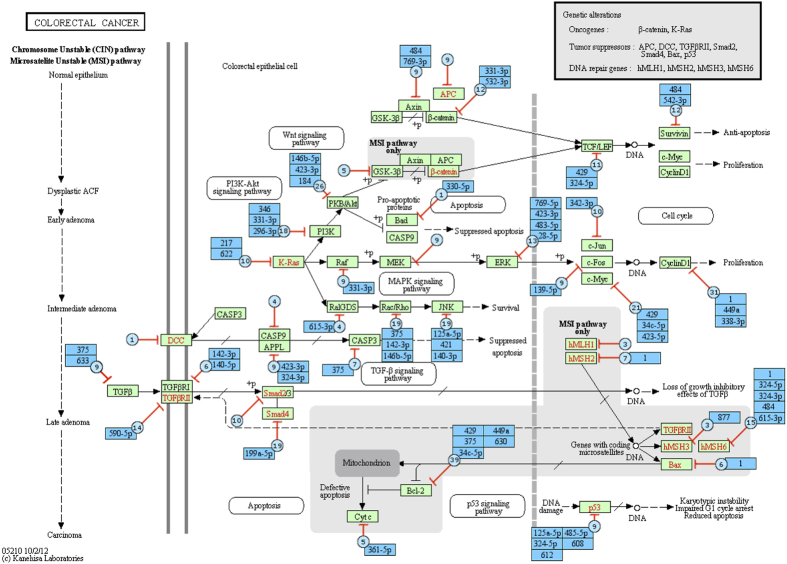
Graphical representation of the augmented pathway *Colorectal cancer*. The green rectangle nodes and black arrows show the KEGG genes and their interactions while the blue nodes and red arrows show the miRNAs and their interactions with the genes, respectively. In each miRNA node added, we show the total number of miRNAs (blue circles) that are known to target the gene, and the names of the miRNA (blue rectangles) that were actually measured in the 8 colorectal miRNA datasets. This is a subset of the total set of miRNAs known to target genes on this pathway.

**Figure 3:**
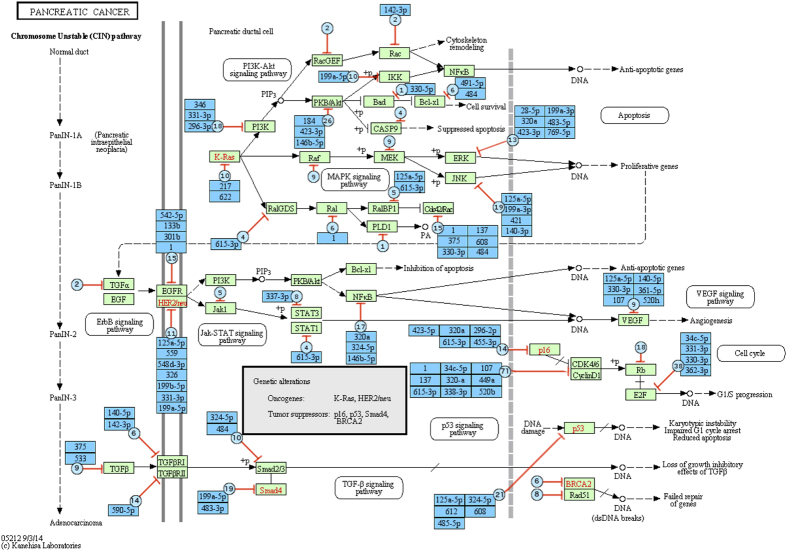
Graphical representation of the augmented pathway *Pancreatic cancer*. The green rectangle nodes and black arrows show the KEGG genes and their interactions while the blue nodes and red arrows show the miRNAs and their interactions with the genes. In each miRNA node added, we show the total number of miRNAs (blue circles) that are known to target the gene, and the names of the miRNA (blue rectangles) that were actually measured in the 6 pancreatic miRNA datasets. This is a subset of the total set of miRNAs known to target genes on this pathway.

For each of the two diseases, we have a *target* KEGG pathway, which is the pathway created to describe the main phenomena involved in the respective disease. The augmented pathway for *Colorectal cancer* is displayed in Fig. 2. The green rectangle nodes show the KEGG genes and the black arrows show the interactions between the genes. The green nodes and the red arrows show the miRNA molecules and their interactions with the genes, where the bar-headed arrow represents the “repression” activity. In each augmented node, we display two types of information: i) the total number of miRNAs that are known to target the corresponding gene, and ii) the miRNAs that were actually measured in the 8 miRNA colorectal datasets. The former is displayed in blue circles while the later is listed in blue rectangles. For example, the gene TGF*β* (in the far left of the figure) has 9 miRNAs that are known to target the gene but only two miRNAs (hsa:miR-375 and hsa:miR-633) were included in the miRNA data. Similarly, the augmented pathway for *Pancreatic cancer* is displayed in Fig. 3. The graphs show that both *target pathways* are heavily regulated by miRNA molecules.

In this experimental study, we expect that a good pathway analysis approach would be able to identify the very pathway that describes the disease phenomena as the most significant in each particular disease. Hence, we will compare the various methods based on this criterion.

### Colorectal cancer

We obtained 8 miRNA (GSE33125, GSE35834, GSE39814, GSE39833, GSE41655, GSE49246, GSE54632, and GSE73487) and 7 mRNA (GSE4107, GSE9348, GSE15781, GSE21510, GSE23878, GSE41657, and GSE62322) datasets from the Gene Expression Omnibus (GEO), as shown in Table 1.

Table 2 shows the results of the 6 approaches. The horizontal line across each list marks the cutoff *FDR* = 0.01. The pathway highlighted in green is the target pathway *Colorectal cancer*. MetaPath_P (pathway-level meta-analysis) identifies no significant pathway at the 1% cutoff, and ranks the target pathway at position 16^*th*^. Similarly, MetaPath_G (gene-level meta-analysis) and MetaPath_I (combination of gene- and pathway-level) identify no significant pathways. They rank the target pathway at positions 9^*th*^ and 15^*th*^, respectively.

The ImpactAnalysis_P approach identifies 12 pathways, among which there are many pathways that are related to cancer. However, the target pathway *Colorectal cancer* is not significant and is ranked 61^*st*^ with adjusted *p* = 0.99. The gene-level meta-analysis (ImpactAnalysis_G) offers some improvement over ImpactAnalysis_P by improving the ranking (10^*th*^) and adjusted p-value (*p* = 0.1) of the target pathway *Colorectal cancer*. However, the target pathway is still not significant with the given threshold. The orthogonal meta-analysis, ImpactAnalysis_I, is able to further boost the power of the gene-level meta-analysis. It identifies 5 significant pathways, with the target pathway *Colorectal cancer* ranked at the very top. This is very likely due to the additional information provided by miRNA expression and prior knowledge accumulated in miRTarBase.

Three of the other 4 pathways that are identified by ImpactAnalysis_I appear to be true positives. The *Cell Cycle* and *Ribosome Biogenesis* pathways are implicated in the proliferation aspect of cancer tissue. *PPAR signaling* has a role in colorectal cancer, although it is not fully understood^59^. *Progesterone-mediated oocyte maturation* is clearly a false positive which may have appeared due to the presence of several cell cycle genes in that pathway.

### Pancreatic cancer

We obtained 8 mRNA (GSE15471, GSE19279, GSE27890, GSE32676, GSE36076, GSE43288, GSE45757, and GSE60601) and 6 miRNA datasets (GSE24279, GSE25820, GSE32678, GSE34052, GSE43796, and GSE60978) from Gene Expression Omnibus (GEO), as shown in Table 1. Again, we compare our approach (ImpactAnalysis_I) with 5 other approaches: pathway-level meta-analysis, gene-level meta-analysis using only mRNA data, plus 3 meta-analysis approaches available in the MetaPath package^30^,^29^ as shown in Table 3.

MetaPath_P identifies no significant pathway and *Graft-versus-host disease* is ranked on top with adjusted p-value 0.4782. The target pathway *Pancreatic cancer* is ranked 17^*th*^ with adjusted *p* = 0.89. MetaPath_G identifies 7 significant pathways. The target pathway is not significant (adjusted *p* = 0.22) and is ranked 91^*st*^. In consequence, the combination of these two methods, MetaPath_I, also fails to identify the target pathway as significant (adjusted *p* = 0.34 with ranking 91^*st*^).

The pathway-level meta-analysis (ImpactAnalysis_P) identifies the *PI3K-Akt signaling pathway* and *MicroRNAs in cancer* as significant. The significance of *MicroRNAs in cancer* may indicate the importance of miRNA in pancreatic cancer, and *PI3K-Akt signaling* alteration is known to be involved in many cancers. However, the target pathway is not significant (adjusted *p* = 0.95 with ranking 32^*nd*^). The gene-level meta-analysis (ImpactAnalysis_G) improves the ranking of the target pathway (8^*th*^) but the p-value of the target pathway is still not significant. The orthogonal approach, ImpactAnalysis_I, identifies 7 pathways as significant. The target pathway *Pancreatic cancer* is ranked on top with FDR-corrected p-value 0.0017.

Of the 6 significant non-target pathways found by ImpactAnalysis_I, three are cancer-related by name (*Small cell lung cancer, Pathways in cancer, Proteoglycans in cancer*). The breakdown of cell matrix adhesions, such as *Focal Adhesion* is an important property of metastasis - most pancreatic cancers are discovered when they are already high grade.

In contrast to the 3 variations of the existing method, MetaPath, the proposed method ImpactAnalysis_I was able to effectively combine both independent datasets, as well as the two different types of data (mRNA and miRNA), and correctly report the target pathway as the most significantly impacted pathway in both meta-analysis studies. The results demonstrate that the correct pathways are identified only when the data are integrated both horizontally (combining multiple studies using the same data type) and vertically (combining miRNA with mRNA expression). This orthogonal meta-analysis uses three different kinds of data integration: integration of mRNA and miRNA, combining p-values and combining SMDs for genes and miRNA molecules.

### Time complexity

The data analysis was done on a personal MacBook Pro that has 8 GB 1600 MHz DDR3 RAM, 2.9 GHz Intel Core i7. Since MetaPath cannot exploit multiple processors, we run all the analysis using a single core. The time needed to run MetaPath was 39 minutes for Colorectal cancer and 47 minutes for Pancreatic cancer.

For ImpactAnalysis_I, we first calculate the p-value for each gene/miRNA in each dataset using the limma package^31^. We then combine the p-values to get one combined p-value per gene/miRNA. Next, we calculate the standardized mean difference (SMD) for each dataset and then apply the REML algorithm to estimate to overall SMD, using the metafor package^56^. The estimated SMDs and the combined p-values are processed by ROntoTools to produce the p-value for each pathway. ImpactAnalysis_I performes the analysis using the pathways augmented with the relevant miRNAs. The running time for ImpactAnalysis_I is 4 minutes for each of Colorectal and Pancreatic. The running time of each approach is reported in Table 4.

## Discussion

One straightforward *horizontal* integration is to combine individual p-values provided by each study. In this way, one can apply any pathway analysis approach (such as GSEA^11^ or GSA^12^) to the collected mRNA datasets in order to calculate a p-value for each pathway in each study, and then combine these independent p-values. The advantage of this approach is its flexibility. MetaPath^30^ combines p-values in this way, but with the slight difference that the p-values are combined on both gene and pathway levels. The drawback is that each of these methods is designed to work with one single matrix of expression values, i.e. one data type. One can forcefully extend this matrix to include other data types as well but in order to do this, the data must be sample-matched. In other words, one must perform all types of assays on every single sample. In addition, since different data types are assayed on different platforms, the data need to be normalized together, for these approaches to function properly. However, the correct way to do such a cross-platform normalization is still an open problem^60^. The same limitations apply to analysis tools dedicated to miRNA and mRNA integration^21^,^61^. For meta-analysis, these approaches would require multiple sets of sample-matched data. Performing different assays on one set of samples is already expensive; asking for many sets of matched samples for the same disease is even more impractical.

Although primarily designed to overcome the matched-sample bottleneck discussed above, our proposed framework also aims to address a well-known limitation of p-value-based meta-analyses. Classical approaches often rely on hypothesis testing to identify differential expression. This results in critical information loss. While the p-value is partly a function of effect size, it is also partly a function of sample size^62^. For example, with large sample size, a statistical test will tend to find differences as significant, unless the effect size is exactly zero. In reality, any individual study will include some degree of batch effects, such as sampling/study bias, noise, and measurement errors. Simply combining individual p-values would not correct such problems. On the contrary, meta-analysis of effect sizes across all studies would definitely compensate for and eliminate such random effects. This point is illustrated in the results included here, in particular in the difference between ImpactAnalysis_P and ImpactAnalysis_G for both colorectal and pancreatic cancer (Tables 2 and 3). The former simply combines the p-values, while the latter takes into consideration both p-values and effect sizes across different studies. ImpactAnalysis_G offers a great improvement over ImpactAnalysis_P using the same sets of mRNA data.

However, the approach proposed here is not without limitations. One such limitation is the computational complexity at both gene and pathway levels. For individual genes and miRNA molecules, the framework not only calculates p-values, but also iteratively estimates the effect sizes and variances. In principle, the iterative algorithm requires more computation than meta-analyses that use closed-form expressions. At pathway-level, Impact Analysis is a non-parametric approach that constructs an empirical distribution of all measured values for each pathway. This requires more computation and storage than parametric approaches, such as the hypergeometric test or Fisher’s exact test. However, this is mitigated by the power of modern computers which are able to perform all needed computations in less than 10 minutes, even for datasets with more than 1,000 samples (Table 4). In addition, our framework allows for parallel computing at the gene-level to reduce the time complexity. However, the time values reported here (in Table 4) do not take advantage of the ability to parallelize the computation in order to be comparable with the results obtained with MetaPath. All values reported in this table are obtained on a single core for both approaches.

The biological results presented here could be further validated by investigating the other pathways reported as significant, and identifying the putative mechanisms that could explain all measured changes. A tool such as iPathway-Guide^63^, could be used to provide more in depth functional analysis, including identification of drugs that are known to act on the observed signaling cascades. Follow-up experiments in which tumor cell lines, or samples from xenografts, are treated with those drugs would validate (or not) both the putative mechanisms investigated, as well as the other significant pathways. If many or all significant pathways were mechanistically implicated in the respective conditions, the proposed orthogonal meta-analysis approach would be further validated.

Another direct application of the orthogonal framework is to infer condition-specific miRNA activity. The proposed gene-level meta-analysis basically identifies genes and miRNAs that are differentially expressed (DE) under the studied condition. This list of DE genes/miRNAs is obtained from a large number of studies and therefore it is expected to be more reliable than any individual study taken alone. From the list of DE genes/miRNAs and the computed statistics (effect sizes and variances), we can identify new putative targets of miRNAs using casual inference techniques^61^,^64^,^65^. The predicted interactions between miRNA and mRNA can be further verified by established gene-specific experimental validation, such as qRT-PCR, luciferase reporter assays, and western blot^66^,^67^.

## Conclusion

In this article, we present a two-dimensional data integration that is able to combine mRNA and miRNA expression data obtained from many independent experiments. The framework first augments pathway knowledge available in pathway databases with miRNA-mRNA interactions from miRNA knowledge bases. It then computes the statistics that are essential for pathway analysis, i.e. the standardized mean difference (SMD) and p-value for differential expression. For each entity, these p-values and the SMDs are computed by combining multiple studies using robust horizontal meta-analysis techniques. Finally, the framework performs a topology-based pathway analysis to identify pathways that are likely to be impacted under the given condition.

To evaluate the framework, we examine 1,471 samples from 15 mRNA and 14 miRNA expression datasets related to two human cancers, using 6 different meta-analysis approaches (3 MetaPath approaches and 3 meta-analysis approaches that utilize Impact Analysis). We demonstrate that the correct pathways are identified only when the data are integrated both horizontally (combining multiple studies using the same data type) and vertically (combining miRNA with mRNA expression).

This work serves as a bridge between the two orthogonal types of data integration. The result is to unblock the sample-matched data bottleneck, by successfully integrating mRNA and miRNA datasets measured from independent laboratories for different sets of patients. Furthermore, it increases the power of statistical approaches since it allows many studies to be analyzed together. With vast databases of various data types being made available, this framework is expected to be widely applicable because of its relaxed restrictions on the data being integrated. The framework is flexible enough to integrate data types other than mRNA and miRNA. It can also be modified to suit other purposes besides pathway analysis.

## Additional Information

**How to cite this article**: Nguyen, T. *et al*. Overcoming the matched-sample bottleneck: an orthogonal approach to integrate omic data. *Sci. Rep.* **6**, 29251; doi: 10.1038/srep29251 (2016).

## Figures and Tables

**Table 1:** Description of miRNA and mRNA expression datasets used in the experimental studies.

Cancer	Data	Accession ID	Control	Disease	Tissue	Platform
Colorectal	mRNA	GSE4107	10	12	Colonic mucosa	Affymetrix HG U133 Plus 2.0
		GSE9348	12	70	Colonic mucosa	Affymetrix HG U133 Plus 2.0
		GSE15781	10	13	Colon	ABI HG Survey 2
		GSE21510	25	123	Colon	Affymetrix HG U133 Plus 2.0
		GSE23878	24	35	Colon	Affymetrix HG U133 Plus 2.0
		GSE41657	12	25	Colonic mucosa, epithelial neoplasm	Agilent-014850 HG 4×44K G4112F
		GSE62322	18	20	Colon	Affymetrix HG U133A
	miRNA	GSE33125	9	9	Colon	Illumina Human v2 MicroRNA
		GSE35834	23	55	Colon & rectum	Affymetrix miRNA 1.0
		GSE39814	9	10	FHC, HCT116, & SW480 cells	Agilent-021827 Human miRNA
		GSE39833	11	88	Peripheral blood serum	Agilent-021827 Human miRNA
		GSE41655	15	33	Colonic mucosa, & epithelial neoplasm	Agilent-021827 Human miRNA
		GSE49246	40	40	Colon	Sun Yat-Sen Human microRNA
		GSE54632	5	5	Colonic and rectal mucosa	Affymetrix miRNA 1.0
		GSE73487	23	90	Colon	Affymetrix miRNA 1.0
Pancreatic	mRNA	GSE15471	39	39	Pancreas	Affymetrix HG U133 Plus 2.0
		GSE19279	3	4	Pancreas, pancreatic duct	Affymetrix HG U133A
		GSE27890	4	4	Pancreas, ductal epithelia	Affymetrix HG U133 Plus 2.0
		GSE32676	7	25	Pancreas	Affymetrix HG U133 Plus 2.0
		GSE36076	10	3	Peripheral blood mononuclear cells	Affymetrix HG U133 Plus 2.0
		GSE43288	3	4	Pancreas	Affymetrix HG U133A
		GSE45757	9	132	Pancreatic epithelial & cancer cells	Affymetrix HG U133A
		GSE60601	3	9	CD14++ & CD16- cells	Affymetrix HG U133 Plus 2.0
	miRNA	GSE24279	22	136	Pancreas	Febit human miRBase v11
		GSE25820	4	5	Pancreatic duct	Agilent-019118 Human miRNA
		GSE32678	7	25	Pancreas	miRCURY LNA microRNA, v.11.0
		GSE34052	6	6	Pancreas	Agilent-029297 Human miRNA
		GSE43796	5	26	Pancreas	Agilent-031181 Human miRNA V16
		GSE60978	6	51	Pancreatic duct	Agilent-031181 Human miRNA V16

All of the data were downloaded from Gene Expression Omnibus.

**Table 2:**
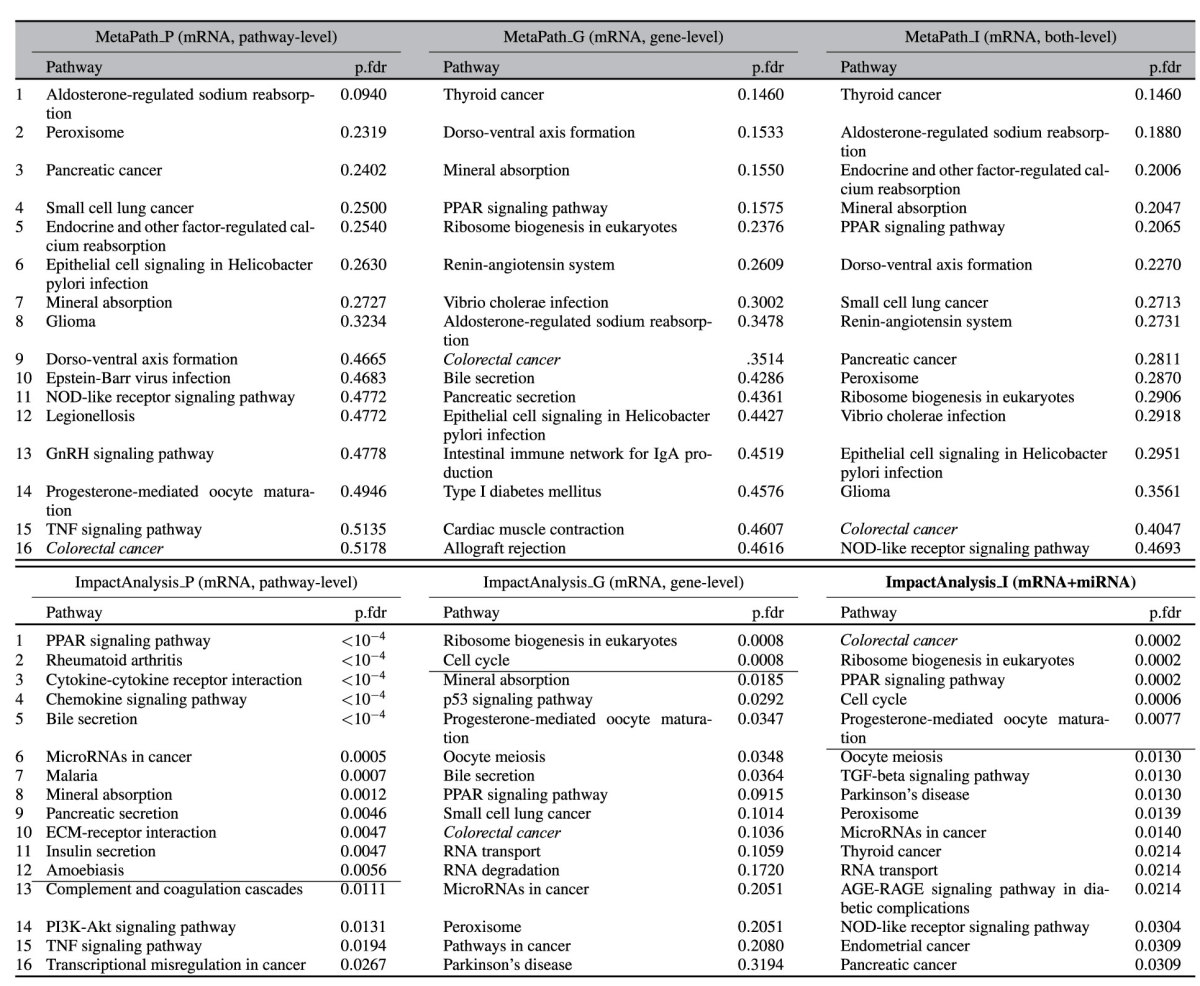
The 16 top ranked pathways and FDR-corrected p-values obtained by combining colorectal data using 6 approaches: MetaPath_P, MetaPath_G, MetaPath_I, ImpactAnalysis_P, ImpactAnalysis_G, and ImpactAnalysis_I.

The horizontal lines show the 1% significance threshold. The target pathway *Colorectal cancer* is *highlighted in green*. All other approaches, MetaPath_P, MetaPath_G, MetaPath_I, ImpactAnalysis_P, ImpactAnalysis_G fail to identify the target pathway as significant, and rank it at the positions 16^*th*^, 9^*th*^, 15^*th*^, 61^*st*^, and 10^*th*^, respectively. On the contrary, the integrative approach, ImpactAnalysis_I, identifies the target pathway as significant and ranks it on top.

**Table 3:**
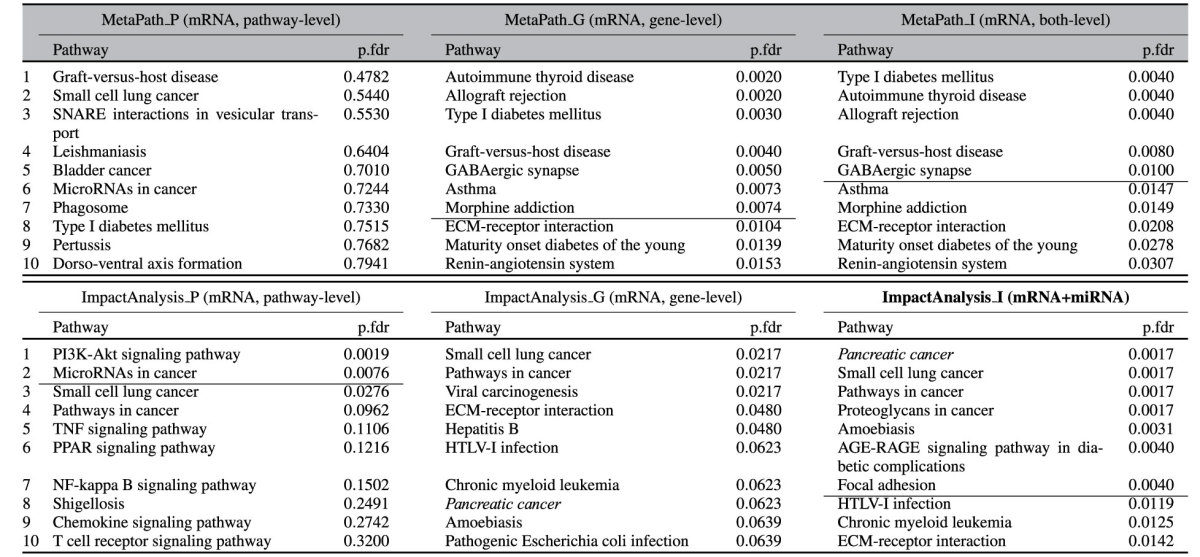
The 10 top ranked pathways and FDR-corrected *p*-values obtained by combining colorectal data using 6 approaches: MetaPath_P, MetaPath_G, MetaPath_I, ImpactAnalysis_P, ImpactAnalysis_G, and ImpactAnalysis_I.

The horizontal lines show the 1% significance threshold. The target pathway *Pancreatic cancer* is *highlighted in green*. All other approaches, MetaPath_P, MetaPath_G, MetaPath_I, ImpactAnalysis_P, ImpactAnalysis_G fail to identify the target pathway as significant, and rank it at the positions 17^*th*^, 91^*st*^, 91^*st*^, 32^*nd*^, and 8^*th*^, respectively. On the contrary, the integrative approach, ImpactAnalysis_I, identifies the target pathway as significant and ranks it on top.

**Table 4:** Running time of each pathway analysis in minutes (m).

Method	Input	Colorectal	Pancreatic
ImpactAnalysis_I	mRNA & miRNA	4 m	4 m
MetaPath	mRNA	39 m	47 m
